# Fibrocartilaginous mesenchymoma of the rib with atypical imaging features

**DOI:** 10.1093/bjrcr/uaaf049

**Published:** 2025-09-18

**Authors:** Rashed Al-Khudairi, Danielle Forster, Sofina Begum, Alexandra Rice, Adrienne M Flanagan, Fernanda Amary, Paul O’Donnell

**Affiliations:** Department of Radiology, Royal National Orthopaedic Hospital, Stanmore, HA7 4LP, United Kingdom; Department of Radiology, Royal National Orthopaedic Hospital, Stanmore, HA7 4LP, United Kingdom; Department of Thoracic Surgery, Royal Brompton Hospital, London, SW3 6NP, United Kingdom; Department of Pathology, Royal Brompton Hospital, London, SW3 6NP, United Kingdom; Department of Pathology, Royal National Orthopaedic Hospital, Stanmore, HA7 4LP, United Kingdom; Research Department of Pathology, Cancer Institute, University College London, WC1E 6DE, United Kingdom; Department of Pathology, Royal National Orthopaedic Hospital, Stanmore, HA7 4LP, United Kingdom; Research Department of Pathology, Cancer Institute, University College London, WC1E 6DE, United Kingdom; Department of Radiology, Royal National Orthopaedic Hospital, Stanmore, HA7 4LP, United Kingdom; Research Department of Pathology, Cancer Institute, University College London, WC1E 6DE, United Kingdom

**Keywords:** fibrocartilaginous, mesenchymoma, bone, tumour, rib, chest, atypical, CT, MRI

## Abstract

Fibrocartilaginous mesenchymoma is a locally aggressive intraosseous lesion first described in 1984, which most commonly presents in the long bones of children and young adults. It is rare—less than 40 cases have been reported to date. The typical radiological features are those of an expansile lytic lesion with chondroid calcification, cortical destruction and frequent extraosseous extension, suggesting an aggressive benign or low-grade malignant tumour. On MRI the lesion returns low T1 and high T2 signal and usually shows intense contrast enhancement. Histologically the lesion is characterised by a spindle cell proliferation, areas of ossification and benign cartilage nodules resembling the epiphyseal plate, the latter considered the hallmark of this tumour. We present a case of fibrocartilaginous mesenchymoma in the rib of a 28-year-old female, a very uncommon location for this tumour. The case is also exceptional because of the age of the patient and its unusual imaging appearances: the lesion showed no evidence of chondral-type matrix mineralisation, displayed profound T2 hypointensity and only minimal enhancement following intravenous Gadolinium administration.

## Clinical presentation

A 28-year-old female presented to her general practitioner with breathlessness on a background of recurrent infections following an episode of COVID-19 infection. The patient reported intermittent pain in the left shoulder, which was exacerbated by deep inspiration and lifting, as well as paraesthesia in the left hand following strenuous activity. There was no palpable mass.

The patient was a non-smoker with no relevant past medical history. The chest was clear on auscultation and oxygen saturation was 100% on room air.

**Figure 1.**
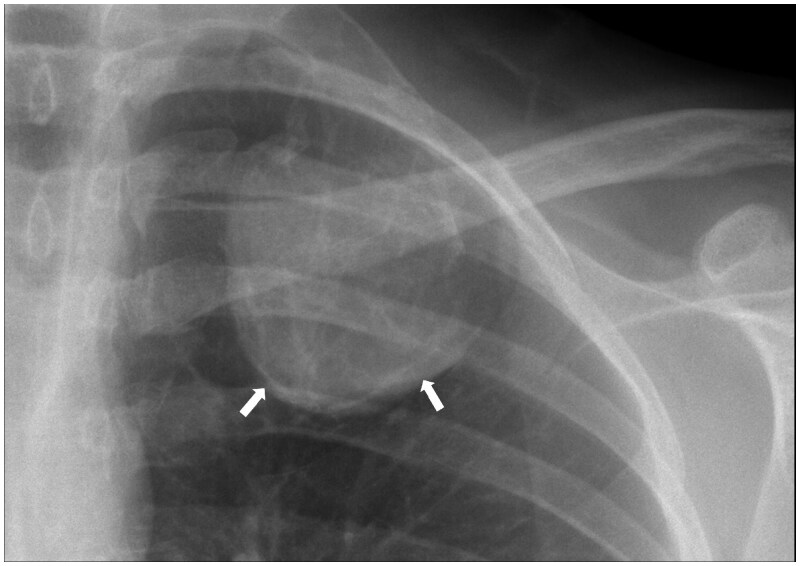
Posteroanterior chest radiograph targeting the left upper zone showing a well-circumscribed peripherally calcified (arrows) expansile lytic lesion arising from the left first rib. Linear densities projected over the mass are also likely to reflect predominantly peripheral mineralisation. The appearances suggest the mass is extrapulmonary.

A chest radiograph was performed which showed a well-marginated, peripherally calcified expansile lytic lesion arising from the left first rib (Figure 1). The patient was referred to a sarcoma centre for clinical assessment and further imaging.

**Figure 2.**
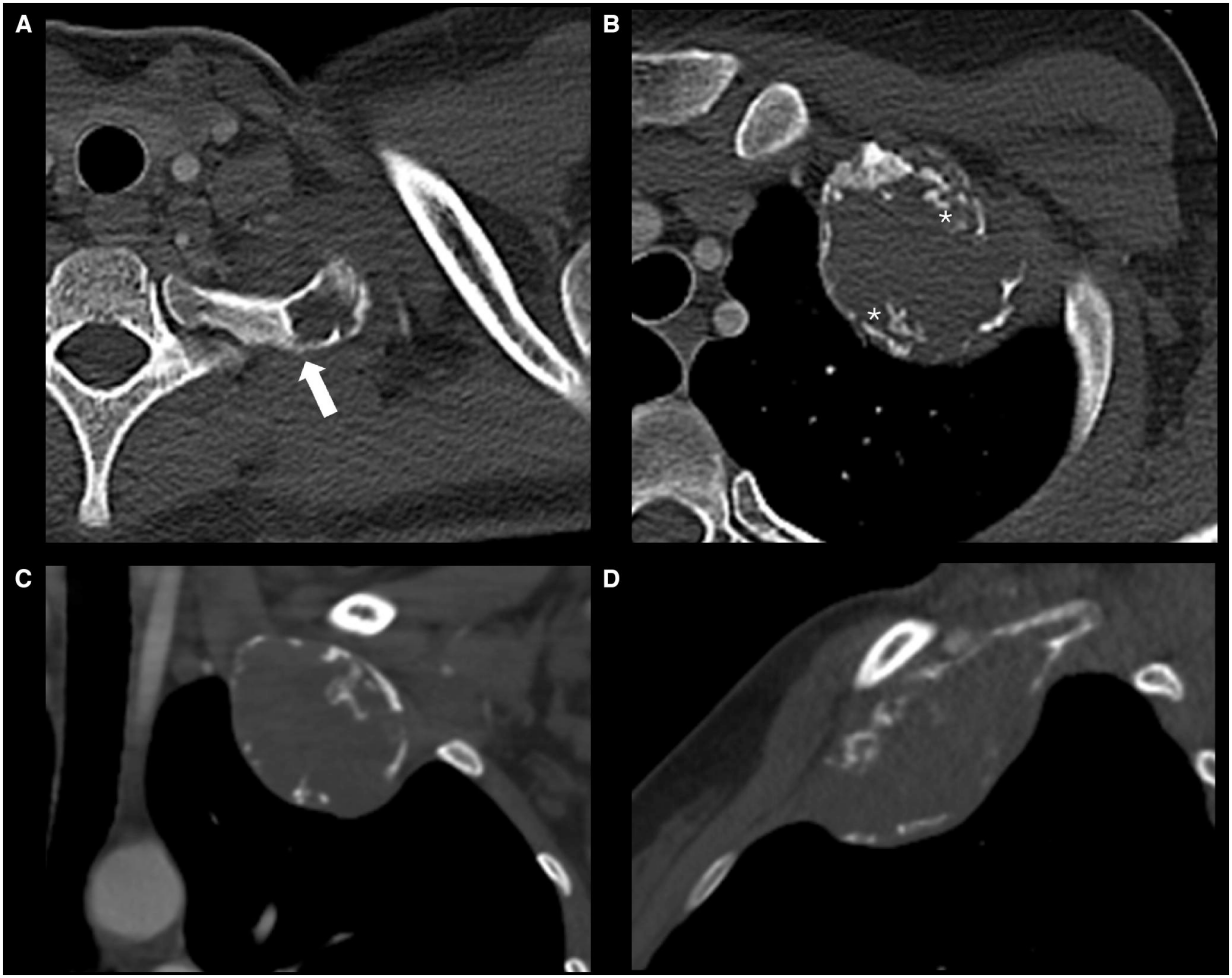
Axial (superior A; inferior B), coronal (C) and sagittal (D) CT images of the left first rib. There is marked expansile remodelling of the rib with anterolateral chest wall deformity. The proximal interface with the bone is not aggressive (arrow, A). The peripheral calcification on radiographs corresponds to irregular cortical thickening; there are intermittent cortical breaches (C). Other calcific foci, the appearances of which suggest curvilinear ossification, are predominantly peripheral (asterisk). Fusiform expansion of the rib is seen (D).

CT of the chest confirmed an expansile lytic lesion involving most of the first rib, sparing the posterior 2.8 cm but reaching the costochondral junction anteriorly (Figure 2). The lesion measured 7.2 cm in the longest (anteroposterior) dimension and demonstrated aggressive features, which included small areas of cortical destruction and subtle extraosseous soft tissue extension: there was intermittent cortical thickening, likely contributing to the radiographic appearances. However, the interface between the lesion and the rib proximally appeared well-defined with a thin sclerotic border, indicative of slow growth. Small irregular and predominantly curvilinear foci of mineralisation, consistent with a bone-forming rather than chondroid matrix, could be seen peripherally, extending towards the centre of the mass. There was no infiltration of, or reactive change in, the adjacent lung despite marked remodelling of the chest wall due to the tumour. There was no other bony abnormality: CT of the abdomen and pelvis was normal.

**Figure 3.**
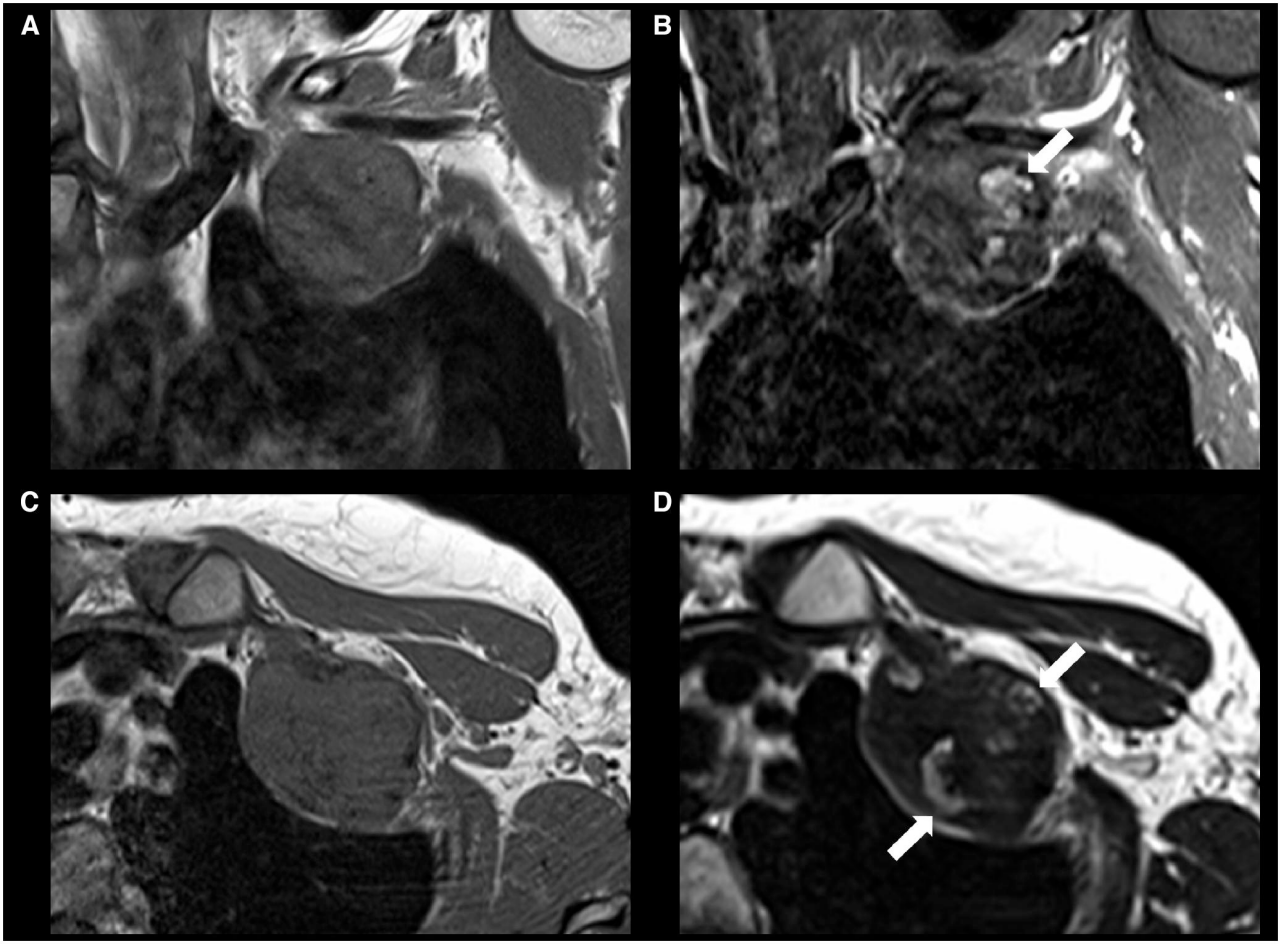
Coronal T1 (A) and STIR (B), axial T1 (C) and T2-weighted (D) MR images of the lesion. It shows heterogeneous low signal intensity on all sequences. There is no perilesional oedema. Lobular hyperintense areas are seen on fluid-sensitive sequences, corresponding to ossified foci on CT (arrows, B and D).

**Figure 4.**
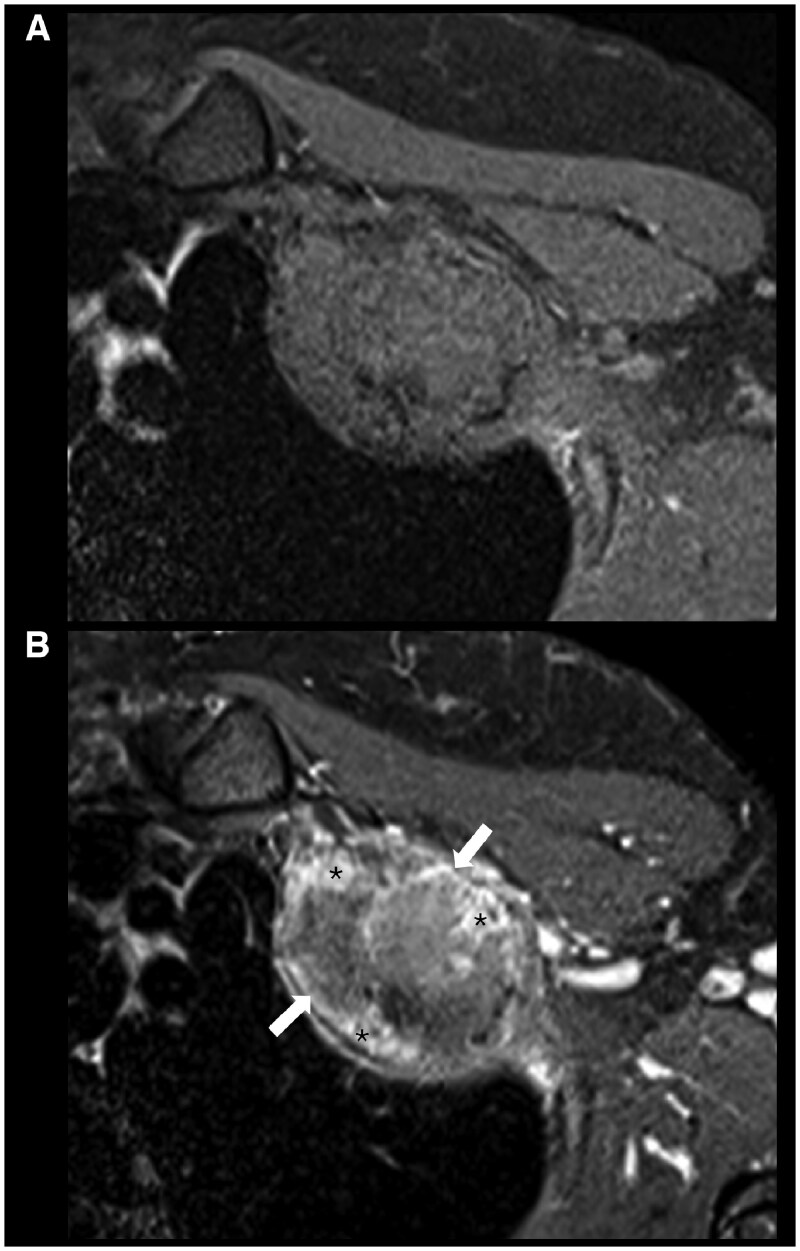
Axial T1 pre- and post-contrast fat-saturated (A, B) MR images demonstrate enhancement of the ossified foci (asterisks) and periphery of the lesion (arrows, B).

On MRI, the lesion demonstrated profound but heterogeneous low signal intensity on all sequences. The predominantly peripheral areas of ossification were hyperintense on fluid-sensitive sequences (T2-weighted/STIR) (Figure 3), and following intravenous contrast administration, moderate enhancement of these foci and the periphery of the mass was seen, with no significant enhancement of the hypointense central component of the tumour (Figure 4). There was no perilesional oedema.

**Figure 5.**
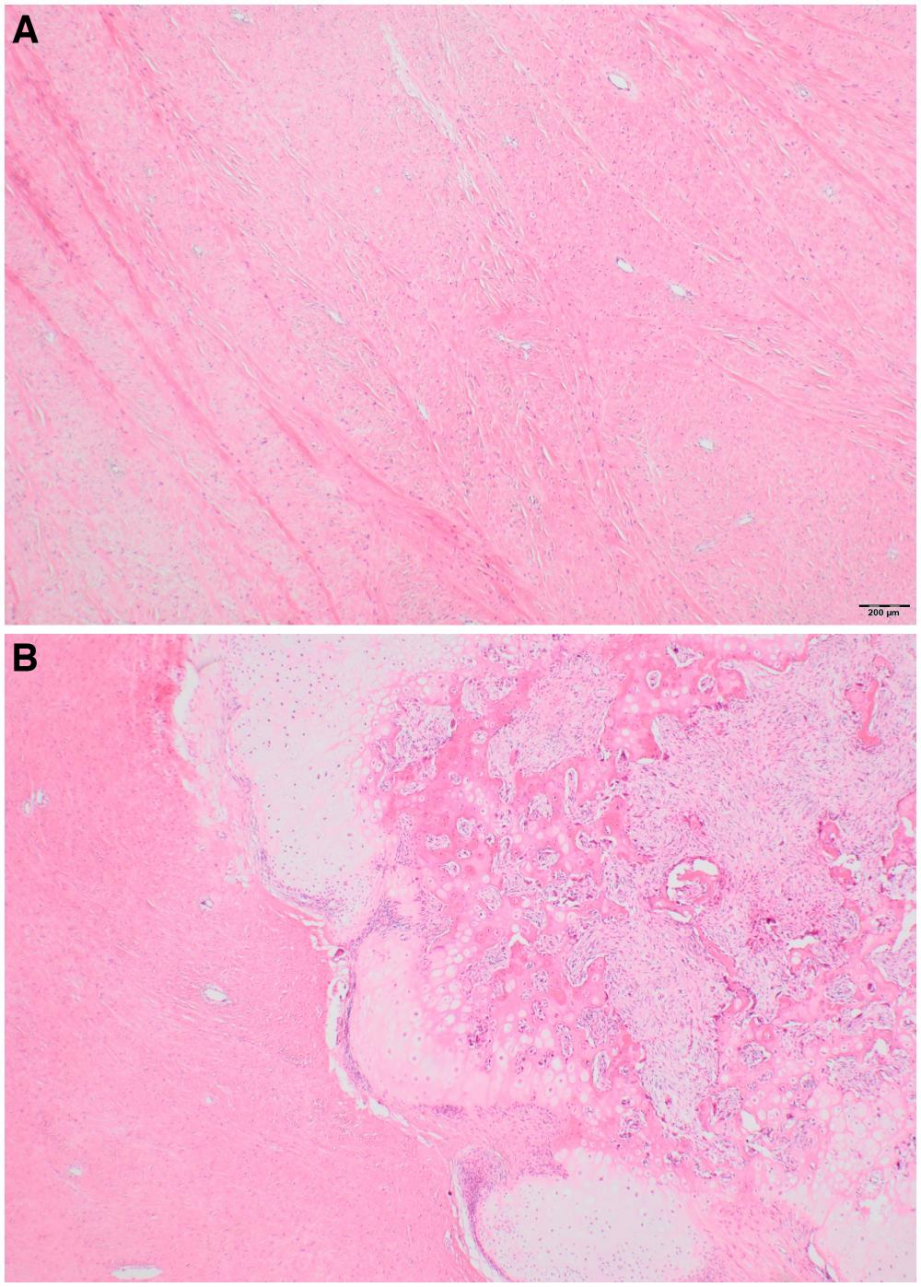
Photomicrographs showing the bland fibrous component (A) and cartilaginous nodule with endochondral ossification simulating the growth plate (B).

These appearances raised the possibility of fibrous dysplasia given the low T2 signal on MRI, but bone destruction was thought to be unusually aggressive. CT guided needle biopsy of the lesion was performed under general anaesthesia. Initial histology suggested a fibrous tumour which was difficult to classify but with no features to suggest malignancy. *GNAS1* mutation analysis was negative for the common point mutations found in fibrous dysplasia. There was no *MDM2* gene amplification by fluorescence *in situ* hybridisation assay, excluding low-grade central osteosarcoma. Subsequently the lesion was excised, and the chest wall was reconstructed with mesh. The resection specimen showed a lesion with fibroblastic, chondroid and osseous elements, simulating the growth plate in areas (Figure 5). The features were in keeping with fibrocartilaginous mesenchymoma. The patient underwent MRI surveillance one- and two-years following surgery with no evidence of local recurrence.

## Discussion

Fibrocartilaginous mesenchymoma (FM) is an extremely rare intra-osseous lesion which was first described by Dahlin et al in 1984.^1^ Histologically, the tumours in this series resembled the epiphyseal growth plate but also had a fibroblastic component that demonstrated hypercellularity, nuclear hyperchromasia and rare mitotic figures, interpreted as low-grade malignant features. FM is currently classified in the intermediate (locally aggressive) category. To date there are less than 40 cases reported in the literature.^2–4^

Most cases of FM have occurred in the long bones of children and young adults, with occasional cases in flat bones and vertebrae: the median age at diagnosis is 13 years, but there is a wide range (three months to 27 years) and a slight male predilection.^2^ Patients usually present with pain, a mass and occasionally a pathological fracture.^1^^,^^2^ FM may rarely be discovered incidentally, for example after an episode of trauma.^5^ In our case, the patient’s symptoms were unlikely to be caused by the mass.

Radiographically, FM is lytic, shows expansile remodelling, cortical destruction and frequent extra osseous extension.^2^^,^^6^ The calcification shows ‘ring and arc’ and ‘popcorn-like’ morphology, typical of a cartilaginous neoplasm.^1^^,^^2^^,^^7^ Most of the case series predate MRI, but one study described FM as returning low signal on T1 and high signal on T2 weighted images^2^^,^^3^^,^^7^ and usually showing strong contrast enhancement.^2^

Our patient presented at quite an old age for this diagnosis (28 years) - the second oldest recorded case. The location of this lesion in the rib is rare (only the third documented case in a rib).^1^^,^^4^ Atypical imaging features included the pattern of matrix mineralisation (peripheral ossification); the signal characteristics on MRI (low signal intensity on all sequences, including the T2-weighted and STIR images, which has not been previously documented) and minimal contrast enhancement, predominantly peripheral and in the ossified areas. Hypointense signal and location in a rib could suggest the diagnosis of fibrous dysplasia, in the absence of the aggressive radiographic features seen in our case. Previous reports have shown wide variability in the pattern of contrast enhancement in FM, ranging from ‘mild, predominantly peripheral’^3^ to ‘partially enhancing’^8^ and massive uptake’.^2^

Radiological differential diagnoses in general include fibrous (such as fibrous dysplasia, fibrosarcoma, desmoplastic fibroma, and chondromyxoid fibroma) and cartilaginous (chondrosarcomas, including clear cell, dedifferentiated and mesenchymal) tumours. Other possible radiological diagnoses include plasmacytoma and giant cell tumour. Mesenchymal hamartoma of the chest wall can resemble FM radiologically and histologically: it occurs almost exclusively in infants, an age group which is only rarely affected by FM. Overlapping imaging features include an extrapleural lesion, an expansile rib mass and mineralisation which is usually chondroid in nature. However, chest wall hamartomas can erode and displace adjacent ribs and show haemorrhagic, peripherally enhancing cystic spaces (secondary aneurysmal changes).^9^

The lesion that most closely resembles FM radiologically is fibrous dysplasia with cartilaginous differentiation (fibrocartilaginous dysplasia), which can be indistinguishable.^2^ However, the radiographic appearances of FM are usually more aggressive, and fibrous dysplasia may be polyostotic. There has been debate as to whether fibrocartilaginous mesenchymoma and fibrocartilaginous dysplasia represent a single tumour entity.^10^ However, the absence of *GNAS* mutation in FM and the subtle differences in the spindle cell proliferation pattern suggest they are distinct histological entities.^2^

Close histological mimics include desmoplastic fibroma and low-grade central osteosarcoma (LGCOS), which can resemble each other if ossification in the latter is inconspicuous. Confirmation of FM from needle biopsy alone can be difficult: a recent case of FM of the pelvis was diagnosed as desmoplastic fibroma due to the lack of cartilaginous nodules.^3^ In our case, the initial needle biopsy showed an indeterminate fibrous lesion, and low-grade osteosarcoma was considered, the correct diagnosis only reached after resection.

In summary, fibrocartilaginous mesenchymoma is an extremely rare intra-osseous lesion, and the rib is one of the most unusual sites. Imaging features can closely resemble fibrous dysplasia with cartilaginous differentiation. The case presented in this report was atypical in lacking chondroid matrix mineralisation, the typical strong post-gadolinium enhancement and showing profound hypointensity on fluid-sensitive MR sequences. Needle biopsy can be misleading if the histological hallmark of the lesion, cartilaginous nodules resembling the epiphyseal growth plate, is inconspicuous.

## Learning points

- Fibrocartilaginous mesenchymoma can rarely present in the rib as a lytic expansile destructive lesion.
- Imaging most closely resembles fibrous dysplasia with cartilaginous differentiation. In our case the lesion demonstrated profound hypointensity on fluid sensitive MR sequences, a paucity of enhancement and lack of chondroid mineralisation. These atypical features have not been previously reported.
- Needle biopsy alone may be misleading if the growth-plate-like, nodular, cartilaginous component is not identified.
